# Coronary Artery Dissection in a Patient with Buerger's Disease

**DOI:** 10.21470/1678-9741-2018-0136

**Published:** 2019

**Authors:** Ali İhsan Tekin, Ümit Arslan

**Affiliations:** 1 Health Sciences University Kayseri Education and Research Hospital, Kayseri, Turkey.; 2 Health Sciences University Erzurum Education and Research Hospital, Erzurum, Turkey.

**Keywords:** Thromboangiitis Obliterans, Myocardial Infarction, Vasculitis, Coronary Artery Disease, Coronary Artery Bypass, Saphenous Vein

## Abstract

Buerger's disease, vasculitis of small and medium-sized blood vessels, is a non-atherosclerotic and progressive occlusive condition which frequently involves the distal part of the limbs. The occlusion of coronary arteries in Buerger's disease is a rare condition; however, coronary artery dissection has not been reported previously. Therefore, this paper presents a 45-year-old man who developed coronary artery dissection associated with Buerger's disease. The patient was treated successfully with coronary artery bypass grafting with the left internal mammary artery to the left anterior descending artery, and saphenous vein graft to the right coronary artery.

**Table:** 

Abbreviations, acronyms & symbols	
BD	= Buerger's disease
CABG	= Coronary artery bypass grafting
LAD	= Left anterior descending
LIMA	= Left internal mammary artery
RCA	= Graft to the right coronary artery
SCAD	= Spontaneous coronary artery dissection

## INTRODUCTION

Buerger's disease (BD), also known as the thromboangiitis obliterans, is a type of vasculitis involving the small- and medium-sized blood vessels and it frequently involves the distal part of the extremities. The disease causes a tightening or blockage in distal limb arteries. It usually appears in middle-aged male smokers, besides disease remissions and relapses are correlated with smoking^[1]^.

BD begins with claudication of the upper and lower limbs. As the disease progresses, superficial thrombophlebitis, Raynaud's phenomena, limb claudication, rest pain, ischemic ulcerations, or gangrene in the distal limbs may also develop. Although coronary artery occlusion in Buerger's disease is a rare condition, to the best of our knowledge, coronary artery dissection has not been described previously yet.

Accordingly, in this paper, we have presented a 45-year-old man who developed coronary artery dissection associated with BD. The patient was successfully treated with coronary artery bypass grafting (CABG) with the left internal mammary artery (LIMA) to the left anterior descending (LAD) artery, and saphenous vein graft to the right coronary artery (RCA).

## CASE REPORT

A 45-year-old man with a previous diagnosis of BD for two years was admitted to the emergency department due to acute chest pain. The patient had no history of diabetes mellitus, hyperlipidemia or hypertension, while he had 25 pack-year history of smoking.

**Fig. 1:**
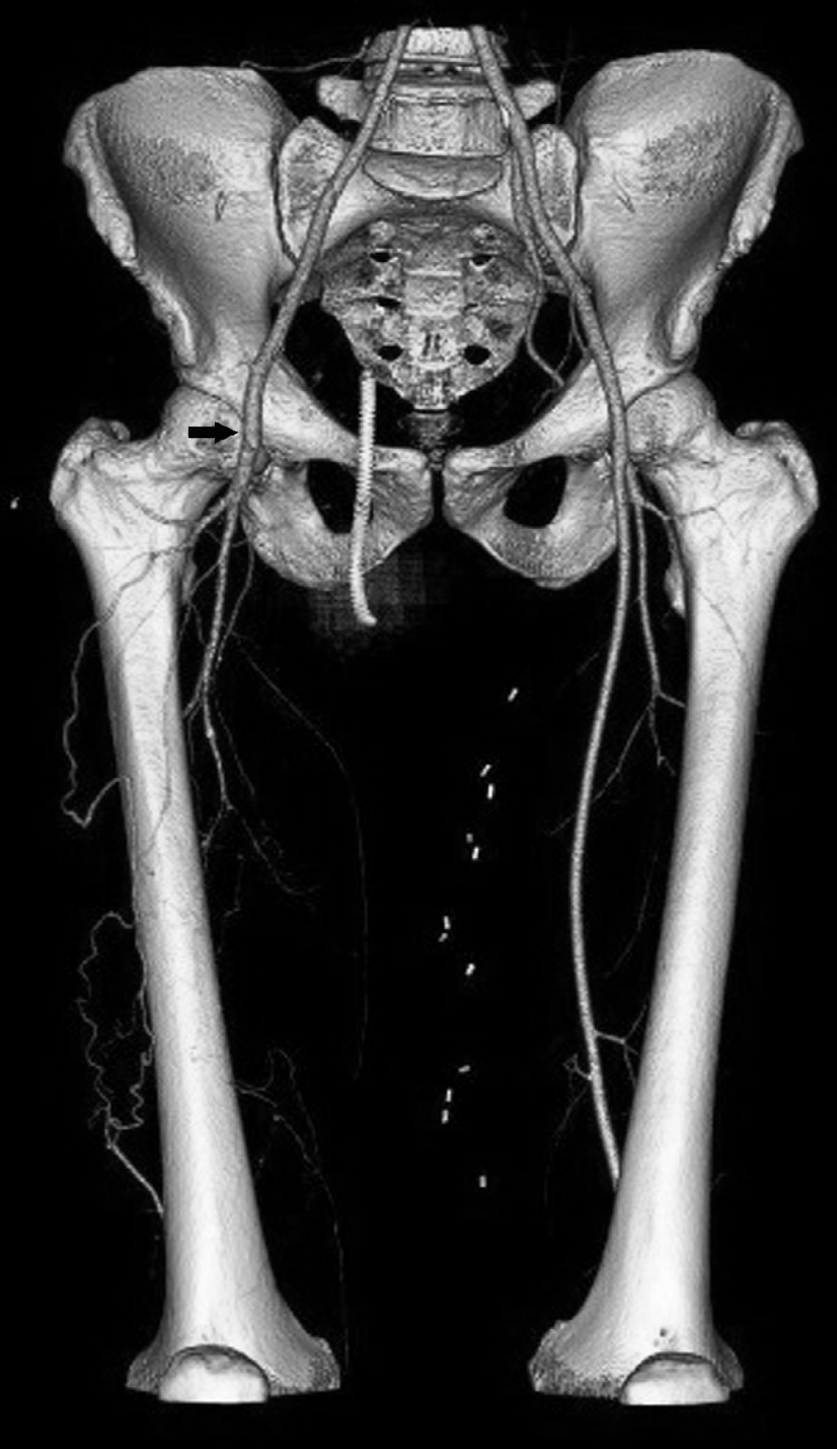
Computed tomography angiography. 3D reconstruction image demonstrates a total occlusion of the right superficial femoral artery (arrow).

**Fig. 2:**
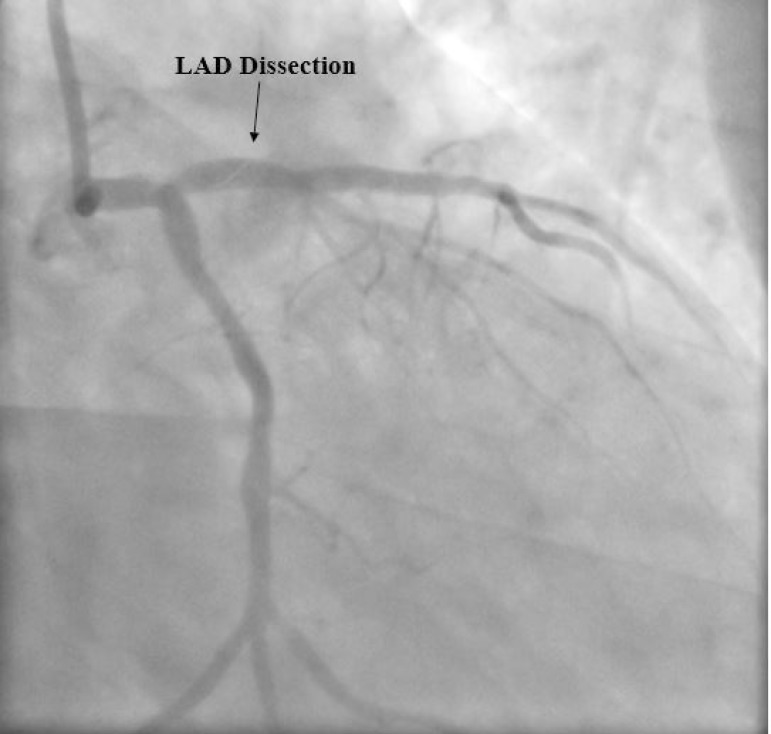
Coronary angiography demonstrates a linear image consistent with coronary dissection (arrow) in the left anterior descending (LAD) artery.

There was total occlusion of the right superficial femoral artery on computed tomography scan (Figure 1). His electrocardiogram revealed a significant ST segment elevation on anterior derivations. Cardiac troponin (7.263 ng/mL, 0-0.1 ng/mL) and creatine kinase-MB (63 U/L, 0-25 U/L) levels were elevated. After the patient was transferred to the coronary care unit with the diagnosis of acute anterior myocardial infarction, an emergent coronary angiography was performed. Coronary angiography demonstrated coronary dissection in the LAD (Figure 2).

**Fig. 3:**
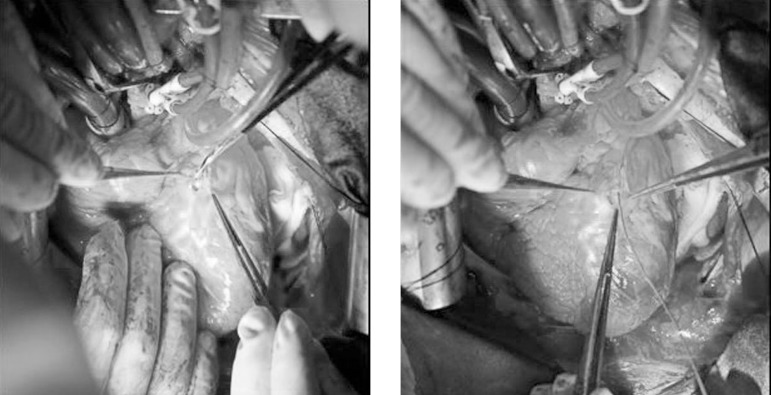
Intraoperative image illustrates the intimal dissection originated from the left anterior descending coronary artery.

Once diagnosed, the patient was taken up for emergency surgery and underwent CABG using the LIMA to LAD and the saphenous vein for RCA surgery. The intimal dissection originated from the LAD was observed intraoperatively (Figure 3). Five days after surgery, the patient was discharged after an uneventful hospital stay.

## DISCUSSION

BD, small- and medium-sized artery inflammation, usually occurs in men from late adolescence to middle age, as in our case. However, recent studies have reported an increasing incidence of BD in women and in people over 50 years of age. The underlying cause of BD is unknown, nonetheless, there is a strong relationship between tobacco use and disease development. BD begins with a decrease in blood flow in the distal small vessels, with progressive involvement of the more proximal vasculature. Studies have reported that the involvement of distal parts of the limbs is much more than other sites, such as coronary, cerebral, intestinal artery and/or vein. Although coronary artery occlusion in BD is very unusual and rarely reported, coronary artery dissection has not been previously described.

When we reviewed the literature regarding coronary involvement, very few reports were found. Hoppe et al.^[2]^ described a 39-year-old female with BD who presented with acute myocardial infarction. Coronary angiogram of the patient revealed obstructive epicardial coronary artery disease. Histological examination of the specimen was consistent with thromboangiitis obliterans. Similarly, Becit et al.^[3]^ reported a 36-year-old man with BD who was treated with CABG. The endarterectomy specimen has shown characteristic findings of inflammatory vasculitis. Moreover, Mautner et al.^[4]^ reported a review of a case series considering the histopathologic evaluation of the coronary arteries whereby atherosclerosis is the predominant histological finding, with the coexistence of lesions consistent with BD. On the other hand, our patient presented with acute myocardial infarction and spontaneous LAD dissection.

Spontaneous coronary artery dissection (SCAD) is a rare condition that can lead to myocardial infarction and even can be fatal. It is defined as a non-traumatic and non-iatrogenic separation of the coronary vessel walls, forming a false lumen^[5,6]^. Although the pathological mechanism of SCAD is still unknown, several clinical conditions, such as atherosclerosis, peripartum period, drugs, heavy exercise, fibromuscular dysplasia, systemic inflammatory disease, and connective tissue disease, have been associated with SCAD^[7]^. To our best knowledge, coronary artery dissection in BD has not been previously described in the literature. In our case, coronary dissection was detected in the LAD artery, and coronary occlusion in the RCA. Regarding the cause for SCAD, presence of segmental vasculitis and increased vascular stress area are plausible hypothesis to explain the coronary artery dissection. The pathological mechanism leading to acute (neutrophilic infiltration involving the thrombus) or chronic (the thrombus with predominantly mononuclear infiltration and fibrosis) inflammation of the vessels may lead to a predisposition to spontaneous dissections in BD^[8]^.

## CONCLUSION

In conclusion, by presenting our unusual case, we would like to draw attention to the fact that coronary artery dissection should be taken into account for the differential diagnosis of acute chest pain in patients with BD. Since the coronary artery dissection leads to sudden death, prompt diagnosis and management are of paramount importance. In this context, CABG seems to be a convenient treatment method, as in our case. Further studies considering the histopathologic changes of the coronary arteries in BD are awaited in order to provide the underlying mechanism of dissection.

**Table:** 

Authors' roles & responsibilities	
AİT	Substantial contributions to the conception or design of the work; or the acquisition, analysis, or interpretation of data for the work; final approval of the version to be published
ÜA	Substantial contributions to the conception or design of the work; or the acquisition, analysis, or interpretation of data for the work; final approval of the version to be published
